# Kinetics of the B-A transition of DNA: analysis of potential contributions to a reaction barrier

**DOI:** 10.1007/s00249-018-1276-4

**Published:** 2018-02-05

**Authors:** Dietmar Porschke

**Affiliations:** Max Planck Institute for Biophysical Chemistry, 37077 Göttingen, Germany

**Keywords:** B-A transition, Kinetics, Magic angle measurements, Electrostatics, Solvent isotope effect

## Abstract

Because of open problems in the relation between results obtained by relaxation experiments and molecular dynamics simulations on the B-A transition of DNA, relaxation measurements of the B-A dynamics have been extended to a wider range of conditions. Field-induced reaction effects are measured selectively by the magic angle technique using a novel cell construction preventing perturbations from cell window anisotropy. The kinetics was recorded for the case of poly[d(AT)] up to the salt concentration limit of 4.4 mM, where aggregation does not yet interfere. Now experimental data on the B-A dynamics are available for poly[d(AT)] at salt concentrations of 0.18, 0.73, 2.44 and 4.4 mM. In all cases, a spectrum of time constants is found, ranging from ~ 10 μs up to components approaching ~ 1 ms. The relatively small dependence of these data on the salt concentration indicates that electrostatic effects on the kinetics are not as strong as may be expected. The ethanol content at the transition center is a linear function of the logarithm of the salt concentration, and the slope is close to that expected from polyelectrolyte theory. The B-A transition dynamics was also measured in D_2_O at a salt concentration of 2.4 mM: the center of the transition is found at 20.0 mol/l H_2_O and at 20.1 mol/l D_2_O with an estimated accuracy of ± 0.1 mol/l; the spectrum of time constants at the respective transition centers is very similar. The experimental results are discussed regarding the data obtained by molecular dynamics simulations.

## Introduction

Looking at molecular models of the B- and A-form DNA suggests that the transition between these well-ordered, right-handed double-helical structures (Saenger 1984) is a relatively simple rearrangement. The molecular details of B- and A-helix structures in crystals have been analyzed at high resolution (Egli et al. 1998; Schneider et al. 1998; Dickerson and Ng 2001; Ng and Dickerson 2002). A short summary of the major differences: (1) the helix increase per base pair is 3.4 Å in the B form and ~ 2.9 Å in the A form; (2) the planes of the base pairs are inclined with respect to the helix axis at angles of ~ 90° for the B and of 74° for the A form; (3) the sugar pucker is C2′-endo for the B and C3′-endo for the A form; (4) the hydration is more extensive in the B form and more “economic” in the A form (Saenger et al. 1986). Dickerson and Ng ( 2001, 2002) concluded from their analysis of the structures that there is a smooth transition between the B and A forms without major activation barriers.

The equilibrium conditions for the existence of the B- and A-form DNA in solution have been characterized in detail (Ivanov et al. 1974). In general, the B form is observed in usual aqueous solutions, whereas formation of the A form requires reduction of the water activity, e.g., by addition of ethanol. Because addition of ethanol not only favors the A form but also aggregation and precipitation of DNA, such reactions had to be avoided by reduction of the salt concentration. These are the general boundary conditions for experimental studies of the B-A transition in solution—obviously valid for studies of both the equilibrium and kinetics. Boundary conditions and the high rate of the B-A transition restrict the experimental potential for analysis of the kinetics. The dynamics of the B-A transition could only be analyzed by the electric field jump technique (Jose and Porschke 2004, 2005). Electric field pulses induce a reaction from the A toward the B form. Experimental evidence (Jose and Porschke 2004) indicates that the A → B reaction is driven by “dipolar stretching;” a dipole increase is expected upon the A → B reaction resulting from an increase of the contour length. A contribution to the driving force may also come from a dissociation field effect (Onsager 1934). The B → A reaction recorded at zero field strength (after electric field pulses) always showed a spectrum of time constants with a major amplitude at ~ 10 μs followed by slower components with time constants not larger than ~ 1 ms.

The dynamics of the B-A transition has been studied more extensively by molecular dynamics simulations than by experiments in solution. Initial simulations suggested that the transition proceeds within ~ 1 ns in both directions. Further simulations with accepted force fields indicated that the transition is observed only in the direction from the A to the B form in aqueous solutions with time constants of ~ 1 ns, whereas the opposite reaction from the B to the A form, expected to occur at reduced water activity, has not been observed under these conditions yet (Cheatham and Kollman 1996; Cheatham et al. 1997; Sprous et al. 1998; Noy et al. 2007; Knee et al. 2008). Meanwhile the conclusion about a low rate for the B-A transition at, for example, 85 volume% ethanol (vol% EtOH) appears to be accepted in general. Apparently, there is a reaction barrier for the B-A transition at reduced water activity. Cheatham et al. (1997) observed a reaction from the B to A form in 85 vol% EtOH when the C3′-endo sugar pucker was stabilized, and Knee et al. (2008) observed the reaction from the B to the A form, when “waters were restrained in the major groove of B DNA.” In summary, the simulations indicate the existence of a reaction barrier under the conditions of reduced water activity.

The present investigation was executed with the goal to obtain more detailed information about contributions to the activation barrier. The experimental data obtained in the preceding investigations were restricted to a limited range of low salt concentrations, which may be a serious problem in particular for a reaction of a polyelectrolyte. Thus, an extension of the data on the B-A dynamics to higher salt concentrations should be useful. Poly[d(AT)] was selected for these tests because its B-A transition appears in a very narrow range of solvent conditions and thus can be characterized at a higher accuracy than for natural DNAs with mixed sequences. Another favorable property of poly[d(AT)] for this analysis is a relatively high solubility at reduced water activity.

Because the B-A transition is induced by reduction of the water activity and hydration of the double helix is essential for the transition, effects upon replacement of H_2_O by D_2_O may provide information about the nature of interactions. Such analysis is also suggested by the possibility “that the barrier for the transition lies in the organization of the solvent” (Knee et al. 2008).

## Materials and methods

Poly[d(A-T)] from Sigma was dialyzed extensively, first against a high salt buffer with 0.2 M NaCl, 1 mM cacodylate pH 7.0, 1 mM EDTA and finally against 250 μM NaCl, 250 μM cacodylate pH 7.0, 50 μM EDTA. Solutions were prepared for measurements by mixing DNA, salt and buffer first; ethanol was added in the last step, except for minor water quantities for filling up to the desired volume. Quantities were controlled by pipetting and weighing. Samples exposed to electric field pulses must be bubble free to avoid cavitation. Thus, these samples had to be degassed under vacuum after filling them into the field jump cells. Because some reduction of the ethanol content cannot be avoided during this procedure, the ethanol content was determined after completion of the field jumps by measurement of the density in a densitometer DMA 602 (Anton Paar, Graz, Austria). UV spectra of the solutions in the cell were recorded directly after filling the cell and after completion of the measurements in a Perkin-Elmer Lambda 17. Conductivities were also determined. The buffers always contained 1 mM Na-cacodylate pH 7 and 0.2 mM EDTA; in addition, there was 1 mM NaCl in buffer A, 3 mM NaCl in buffer B and 10 mM NaCl in buffer C. All field jump experiments were conducted at 2 °C to avoid field-induced denaturation of poly[d(A-T)].

The potential effect of hydrogen/deuterium replacement was tested in solutions prepared in buffer A in H_2_O initially. Then, H_2_O was evaporated—finally residual H_2_O was removed in vacuo over phosphorus pentoxide. Subsequently, the components were dissolved in the required volume of D_2_O, and finally EtOD was added. D_2_O was from Sigma Aldrich, EtOD from Deutero GmbH.

Processes induced by electric field pulses were measured in a device originally constructed by Grünhagen (1974). The main parts of the high-voltage pulse generator are still as described by Grünhagen (1974), whereas other components such as the light source, measuring cell and detector were replaced. The light source used in the present experiments was a 200-W high-stability L2423 Hg/Xe arc-lamp from Hamamatsu together with a Schoeffel GM250 grating monochromator and a Glan air polarizer. A homemade photomultiplier detector was used together with a Tektronix DSA 601A digitizer for data collection. The data were evaluated by a set of previously described programs (Diekmann et al. 1982; Porschke and Jung 1985).

Densities required for conversion between the different units used to define the composition of water-ethanol mixtures were taken from Haynes and Lide (2015) and from “International alcoholometric tables” (https://www.oiml.org/en/files/pdf_r/r022-e75.pdf). Densities for D_2_O-EtOD mixtures were measured by the DMA 602 densitometer (Anton Paar, Graz, Austria).

### Conditions for magic angle detection and construction of improved cell windows

**Fig. 1:**
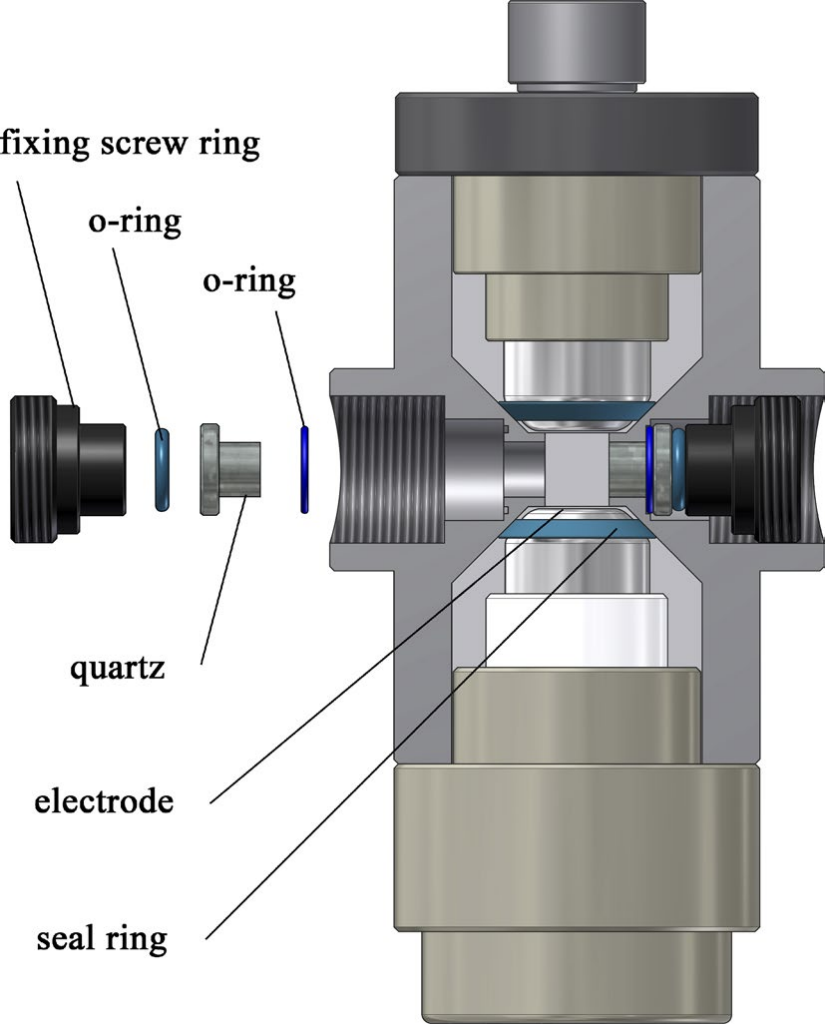
Construction of the cell used for exposure of samples to electric field pulses. Form and dimensions of the cell body (macrolon) and the electrodes (platinum) are essentially as used previously. A major improvement is based on the construction of the cell windows: explosion view (left) and assembled view (right). The windows are not in direct contact with the cell body: the quartz cylinder is held at an outer segment of larger diameter between elastic O-rings (soft silicon rubber), such that the inner segment is “free” in a bore hole adjacent to the sample space

Separation of field-induced reaction effects from orientation effects requires measurements at the magic angle, which is not trivial and demands special experience. The “magic” angle refers to an orientation of polarized light at 54.8° with respect to the vector of the applied electric field (Labhart 1961). Theory and experiments demonstrate that reaction effects are observed selectively at the magic angle (Porschke 1974, 1996). In practice, amplitudes should not exceed ~ 10% of the total light intensity, because magic angle conditions may be violated otherwise. Changes in the light intensity resulting from orientation are usually much larger than those from reactions such as conformation changes; thus, magic angle conditions must be rigorously adhered to. Polarized light at the magic angle can be generated relatively easily, but the state of polarization may be seriously affected by strain in the cell windows. Such strain is induced by any mechanical stress at the optical windows. Quartz is convenient for measurements in the UV, but minor stress may cause a large birefringence, resulting in changes of the polarized light. In the past, birefringence of quartz windows was avoided by a layer of silicon grease between the cell body and the window (Porschke 1996). The viscosity of the grease had to be low enough to avoid mechanical coupling with the cell, but high enough to avoid smearing out into the cell and on the surface of the windows. In practice, cell windows had to be cleaned and reinserted frequently. These problems are avoided by a novel form of window construction and insertion into cell bodies developed recently for extension of birefringence measurements to a particularly high sensitivity (Porschke 2011). This mode of construction also proves to be very useful for absorbance measurements in field jump cells. Because arc lamps are still favorable for measurements in the UV and high light intensities are required for high time resolution, the window dimensions had to be increased. The construction used for the present measurements is shown in Fig. 1. The essential conditions for optimal implementation are: (1) selection of quartz windows completely free of strain; (2) the quartz windows are held at their outer cylinder planes by flexible O-rings made from soft silicon rubber and are not in direct contact with the cell body. Cells with optical path lengths of 7 mm (Fig. 1) and 20 mm (not shown) were used in the present investigation.

## Results

### Salt dependence

**Fig. 2:**
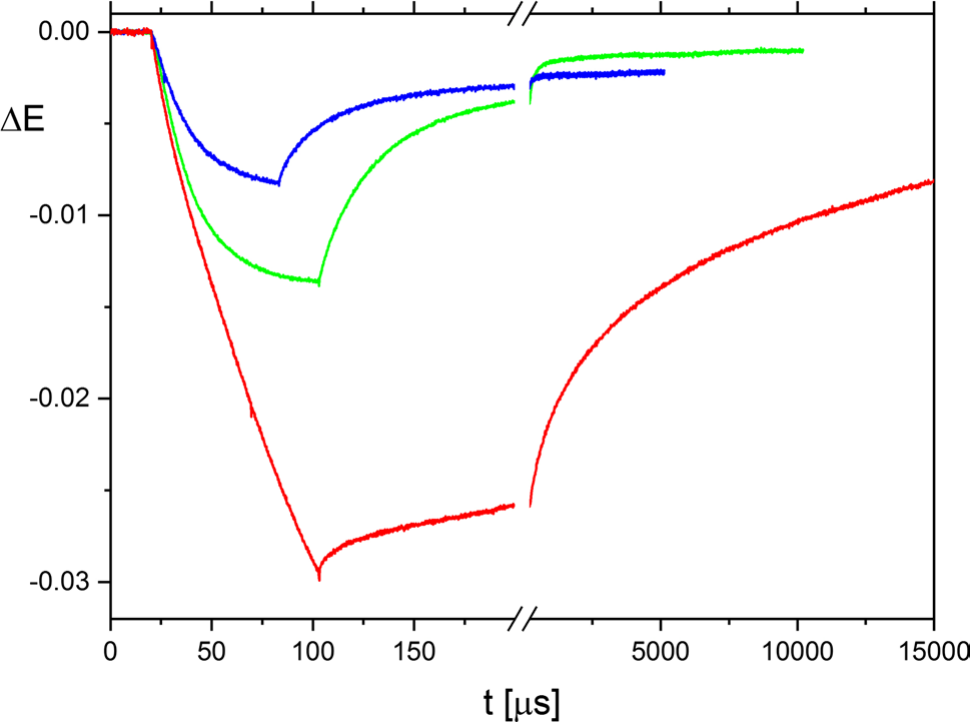
Transients induced by application of electric field pulses to solutions of poly[d(AT)] at different salt concentrations: 2.44 mM at 67.5 vol% EtOH (buffer A, green line), 4.44 mM at 66.71 vol% EtOH (buffer B, blue line) and 11.4 mM at 65.96 vol% EtOH (buffer C, red line). The measured changes of light intensity are converted to extinction changes, which are then normalized to a concentration of 80 μM phosphate units. Electric field pulses start at *t* = 20 μs; pulse lengths are 83, 63 and 83 μs in buffers A, B and C, respectively; pulse amplitudes are 39 kV/cm

Poly[d(AT)] samples in water–ethanol mixtures at specified buffer concentrations were subjected to DC electric field pulses, and the absorbance changes at the magic angle were recorded. As described previously (Jose and Porschke 2004, 2005), the relative absorbance changes associated with the B-A transition are particularly high at wavelengths ≥ 290 nm. For convenient magnitudes of absolute changes in this spectral range, DNA concentrations in the range of ~ 20 to ~ 280 μM (monomer units) were used. Examples of field-induced transients in the range of the B-A transition are shown in Fig. 2 for the buffers A, B and C (green, blue and red line, respectively) at ethanol contents close to the centers of the B-A transition. The shape of the transients and the time constants are similar in buffers A and B. The reduction of the amplitude in buffer B compared to buffer A reflects the increased electrostatic shielding resulting from the increase of the ion concentration in buffer B. A corresponding dependence was observed at the lower salt concentrations used in the previous investigation (Jose and Porschke 2004).

A detailed discussion of the processes observed upon application of the electric field and after pulse termination has been presented previously (Jose and Porschke 2004). Transients measured close to the center of the B-A transition represent large perturbations induced by the field pulses. Clear differences are observed in the reaction progress curves upon application of electric field pulses and after pulse termination. Here, discussions are restricted to reaction curves recorded after pulse termination (i.e., at electric field strength zero), representing a net reaction flow from B to A DNA. These transients show a spectrum of times with a relatively broad range of time constants. Thus, individual time constants cannot be defined as usual, and only approximate numerical values can be given. This is partly because the B-A transition is a cooperative reaction. Fitting of the transients showed a spectrum with a first process at ~ 10 μs followed by slower components with time constants not exceeding ~ 1 ms.

The transient in buffer B indicates a shift in the ∆*E* level after the field jump relaxation with respect to that before application of the electric field (cf. Fig. 2). Analysis of the field jump data and comparison with measurements of the absorbance as a function of the temperature in a standard spectrophotometer demonstrate that the shifts are due to a field-induced temperature jump. This effect is minimal in buffer A because of its low conductivity, but is clearly detectable in buffer B because of its increased salt concentration.

A further increase of the salt concentration in buffer C leads to a clear change of the transient with respect to those found in buffers A and B: (1) the amplitude is much higher, although electrostatic shielding is increased; (2) the response time constants are much larger. These changes in the transient are accompanied by the appearance of turbidity. All these observations indicate the onset of aggregation.

The limiting value of the ethanol content, where aggregation is initiated, decreases with increasing salt concentration. At salt concentrations up to 4 mM, the B-A transition is observed before the onset of aggregation, whereas the B-A transition and aggregation appear at closely corresponding ethanol contents in 10 mM salt.

The aggregation is not only reflected in the response to electric field pulses, but is also indicated by an increase of the absorbance due to light scattering. This effect is clearly visible, for example, at long wavelengths outside the usual absorbance band, already at 63.6 vol% EtOH in the buffer with 10 mM salt, whereas a minor increase was found at 63.0 vol% EtOH. At 4 mM salt, there is no increase of the absorbance in the long wavelength range in the B-A transition range resulting from turbidity; a minor increase was observed above the B-A transition at 68.23 vol% EtOH and a DNA concentration of 260 μM (monomer units). This effect was not visible at a DNA concentration of 72.6 μM (monomer units) and 68.04 vol% EtOH content. Both EtOH content and salt concentration are particularly important parameters for aggregation. In addition, the onset of aggregation is also affected by the DNA concentration.

**Fig. 3:**
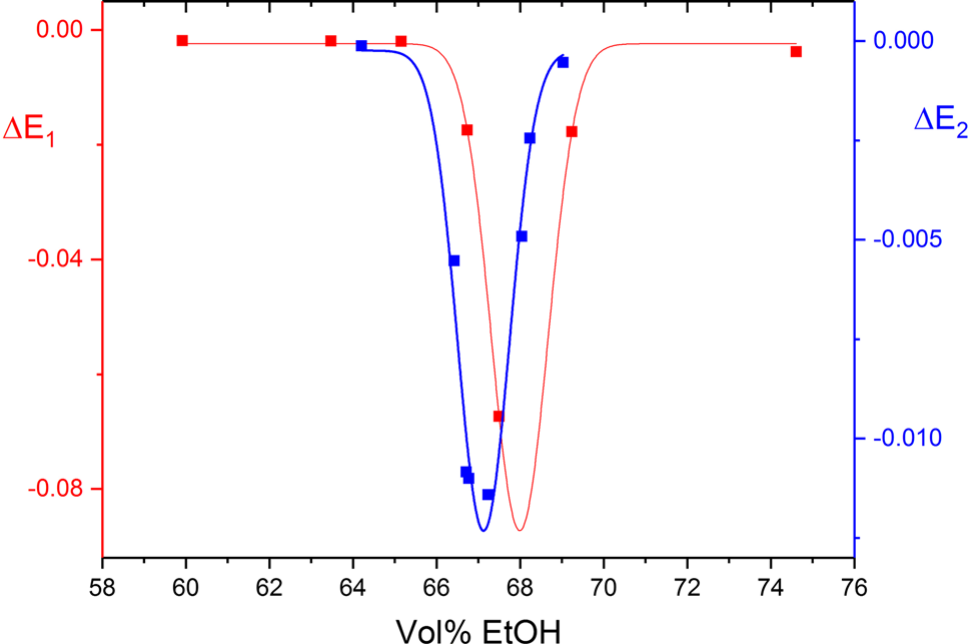
Transition amplitudes induced by electric field pulses of fixed amplitude and duration in poly[d(AT)] as a function of the ethanol content at salt concentrations of 2.44 mM (data points and ∆*E* scale in red, buffer A) and 4.44 mM (data points and ∆*E* scale in blue, buffer B). The data are fitted to Gaussians providing the transition centers 67.99 and 67.12 together with the widths of the transition 1.59 and 1.46 for the buffers A and B, respectively. ∆*E* values normalized to a concentration of 80 μM phosphate units

The field-induced change of the absorbance is maximal at the center of the B-A transition and decays to very small values outside the transition range. The dependence of the amplitudes on the ethanol content is fitted to Gaussians, providing the center and width of the transition (Fig. 3). The amplitudes were measured at different poly[d(AT)] concentrations and were normalized for fitting to a standard concentration of 80 μM. An extreme case is included in the data set at 4.44 mM salt: one of the points (at 66.707 vol% EtOH) was measured at a poly[d(AT)] concentration of 279.6 mM and another one (at 66.775 vol% EtOH) at 23.1 μM. Although these data points were taken at widely different poly[d(AT)] concentrations, the ∆*E* values normalized to 80 μM are very close to each other (Fig. 3). This is further evidence against aggregation effects in the BA transition range of poly[d(AT)] at 4.44 mM salt.

**Fig. 4:**
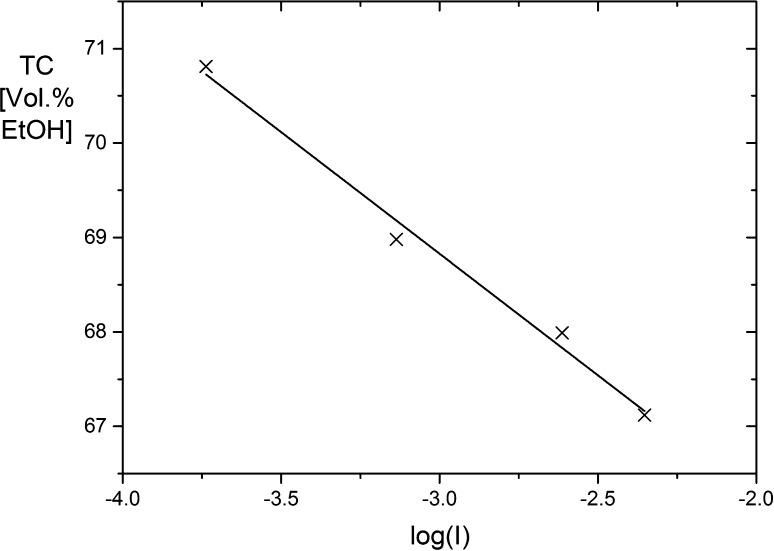
Centers of the B-A transition of poly[d(AT)] as a function of the logarithm of the salt concentration. The slope obtained by linear regression is − 2.57

There are now data for the B-A transition obtained by the electric field jump method at four different salt concentrations ranging from 0.183 to 4.44 mM [data from the present investigation and from Jose and Porschke (2004)]. The ethanol content at the center of the transition is a linear function of the logarithm of the salt concentration (Fig. 4). It has been suggested that the ethanol content can be used as a scale of the free energy at least approximately. Based on the assignments presented by Ivanov et al., changes in the vol% EtOH scale may be translated to free energy changes. The slope obtained by linear regression of the data in Fig. 4 is − 2.57, corresponding to a change of vol% EtOH by 2.57 upon a change of the salt by a factor of 10. Using the free energy changes evaluated for three different decamer helices together with respective changes on the trifluoroethanol volume% scale (Minchenkova et al. 1986), (vol% TFE) provides a correspondence of ~ 235 cal per decamer for 1% change on the vol% TFE scale. Each decamer helix involves nine base stacks.

Thus, a change of the salt by a factor of 10 in the case of poly[d(AT)] corresponds to ~ 70 cal per mole of base stack. The change of the polyelectrolyte free energy ∆G for the B-A transition of double helical DNA was calculated by Manning (2002) as a function of the salt concentration *c*_*i*_: when *c*_*i*_ is decreased from 10^−2^ to 10^−3^ M, ∆G is increased by ~ 100 cal per mole base pair. Because of the approximations involved in the comparison, an exact agreement should not be expected. It may be concluded that the orders of magnitude of the experimental result and of the theoretical prediction are consistent.

### H_2_O/D_2_O exchange

The test for any dependence of the B-A transition parameters upon an exchange of the solvent from H_2_O to D_2_O was performed in buffer A, because the field-induced amplitudes are still favorable under these salt concentration conditions. Field-induced amplitudes were measured as a function of the EtOH and EtOD content, respectively. In both cases, distinct maxima of the amplitudes were observed at the center of the B-A transition. Fitting of the data by Gaussians showed maxima at 67.7 vol% EtOH in H_2_O and at 69.3 vol% EtOD in D_2_O. The estimated accuracy is ± 0.2 vol%. The corresponding procedure applied to the same amplitude data but using a scale based on molar concentrations of H_2_O and D_2_O provided maxima at 20.0 and 20.1 mol/l, respectively, with an estimated accuracy ± 0.1 mol/l. Thus, the center of the B-A transition appears at a slightly higher D_2_O than H_2_O value on the mol/l scale, but the difference is within the limit of accuracy.

Because of the sharp maximum of the amplitudes at the transition center, this center can be determined at a relatively high accuracy. This advantage does not exist for the time constants. Moreover, a special problem appears because of multiexponential transients. Separation of multiexponentials is notoriously difficult; thus, exact values of individual time constants cannot be specified. Under these conditions, the conclusion is limited to the statement that the time constants are very similar in H_2_O and D_2_O.

## Discussion

As demonstrated by high-resolution structures of protein DNA complexes, the B-A transition of DNA is induced upon binding of regulatory proteins to DNA in many cases (Jacobo-Molina et al. 1993; Kiefer et al. 1998; Cheetham and Steitz 1999; Jones et al. 1999; Lu et al. 2000). Thus, the B-A transition is directly involved in the mechanism of gene regulation. An unambiguous assignment of the steps contributing to this biologic function requires sufficient information about the B-A transition.

### Comparison of experimental and simulated results

There are many publications on molecular dynamics simulations from different authors. Because of initial problems in the early phase of simulations, some conclusions about the B-A dynamics were not apparent from the beginning. Fast conversion of A-DNA to B-DNA within ~ 1 ns in aqueous solutions has been simulated in many cases, whereas the opposite reaction from B- to A-DNA, expected to occur at reduced water activity, has not been observed in unbiased MD simulations (Cheatham et al. 1997; Sprous et al. 1998; Noy et al. 2007). Apparently, there is a considerable activation barrier at reduced water activity. This result may be considered consistent with the experimental data obtained at reduced water activity by the electric field jump technique (Jose and Porschke 2004, 2005) in the sense that both experiments and simulations show the existence of activation barriers at reduced water activity. However, there is no explicit information on the time scale of the B-A reaction at reduced water activity from molecular dynamics. Conversely, the field jump technique does not provide information on the B-A kinetics in usual aqueous solutions. It is remarkable that molecular dynamics simulations indicate the existence of a large activation barrier at reduced water activity and the absence of this barrier in usual aqueous environments. This contrast raises questions about the nature of the activation barrier.

The B-A transition is a special case of a stacking rearrangement, which is a frequently observed reaction in nucleic acids. A simple example is single-strand stacking, which has been studied for various cases by cable temperature jump measurements (Porschke 1976, 1977). Time constants in the range of ~ 10 ns to ~ 1 μs were observed. Corresponding results were found by laser temperature jump measurements (Dewey and Turner 1979; Freier et al. 1981). The time constant of stacking in a model compound with two adenine residues connected by a simple and flexible aliphatic –(CH_2_)_3_-bridge could not be resolved by the cable temperature jump technique (Porschke 1978); thus, its stacking time constant is faster than ~ 10 ns. This result indicates that the “activation barrier” in the case of single-stranded stacking is imposed by the ribose- or deoxyribose-phosphate backbone. Stacking is slowed down even further in more complex structures. In simple nucleic acid loops, for example, the rearrangement of stacking was found to occur with time constants ≥ 10 μs (Bujalowski et al. 1986; Menger et al. 2000). All these time constants for simple stacking rearrangements were characterized in aqueous salt solutions. Thus, the time constants observed for the B-A transition by relaxation measurements at reduced water activity are in a time range that may be expected from the time constants of usual stacking rearrangements in a usual aqueous environment. From this point of view, there is no problem regarding the nature of an activation barrier during the B-A transition.

### The aggregation problem

One of the problems associated with the experimental analysis of the B-A transition has always been the decrease of DNA solubility at reduced water activity. Precipitation has been avoided simply by reduction of the salt concentration. However, the criteria for the absence of aggregation have not always been presented clearly enough. Minchenkova et al. (1986) reported that precipitation can be avoided by using trifluoroethanol (TFE) instead of ethanol for reduction of the water activity. A detailed analysis based on electro-optical measurements of rotational diffusion for natural DNA restriction fragments with mixed AT/GC sequences has been presented recently (Porschke 2016), showing a range without aggregation or condensation upon addition of ethanol at monovalent salt ≤ 1 mM. This range for analysis of the B-A transition without aggregation or condensation is extended to monovalent salt ≤ 4.4 mM upon addition of TFE.

In the present investigation, the special effect of TFE was not used. Instead it was tested, how far the set of data available for poly[d(AT)] in H_2_O-EtOH mixtures could be extended. The results show that an extension is possible up to a salt concentration of 4.4 mM. Strong perturbations resulting from aggregation were observed at 11.4 mM salt concentration. The aggregation tendency of DNA without GC base pairs is expected to be reduced. The chain length may be another parameter affecting aggregation. Finally, the kinetics of aggregation should be considered, which is expected to be accelerated with increasing salt and ethanol content. Intermediate experimental conditions of salt and ethanol content are expected to exist where aggregation cannot be avoided at long times but remains negligible at sufficiently short times. Under these conditions there are time windows that can be used for analysis of the B-A transition.

### Magnitude of the electrostatic barrier

An obvious candidate for a contribution to an activation barrier is electrostatic repulsion. The experimental analysis by measurements of the ionic strength dependence has now been extended to a wider range from 0.183 to 4.44 mM. The change of B-A transition time constants in this range is relatively small, indicating that the electrostatic contribution to the reaction barrier is not as large as may have been expected. The experimental time constants have always been determined close to the center of the transition. For each of the analyzed salt concentrations there is a strong dependence of the time constants on the EtOH content. The magnitude of the time constants observed under the impact of the electric field is always reduced with decreasing EtOH contents (Jose and Porschke 2004). However, extrapolation of these dependences to the absence of EtOH is not practicable, because the interval available for measurements is very limited; thus, extrapolation over the wide range of EtOH content to the absence of EtOH cannot be sufficiently accurate. Moreover, the effect of the electric field must be extrapolated as well. Under these conditions, the available experimental data can only be used with sufficient reliability for the conclusion that the time constants at the center of the transition are almost independent of the salt concentration. Because the overall activation barrier corresponds to a factor of ~ 10^4^, the present results indicate that the main part of this barrier is not due to electrostatics.

### Solvent isotope experiment

The H_2_O/D_2_O exchange was conducted as a simple test for an influence of the hydrogen bonding and hydration network on the thermodynamics and kinetics of the B-A transition. The experimental data demonstrate that the B-A transition is observed at a slightly higher value on the mol/l scale for D_2_O than H_2_O, but the difference is within the limit of accuracy. Thus, the effect of the H_2_O/D_2_O exchange on the B-A transition is relatively small. Large effects resulting from an H_2_O/D_2_O exchange have been reported in the literature for protein stability, dynamics and association (Cioni and Strambini 2002; Sasisanker et al. 2004; Eginton and Beckett 2013). Recent simulations on a RNA hairpin (Pathak and Bandyopadhyay 2017) indicate a large effect induced by an H_2_O/D_2_O exchange on its thermal stability. Considering the well-documented strong influence of solvation on the B-A transition (Saenger 1984; Saenger et al. 1986), the absence of a detectable influence of the H_2_O/D_2_O exchange is remarkable and should be analyzed in more detail.

## Conclusions

An improved construction of optical windows for electric field jump cells is developed for selective characterization of field-induced reactions in the presence of orientation effects by magic angle measurements. Extension of the experimental data on the kinetics of the B-A transition in poly[d(AT)] to a wider range of salt concentrations indicates that electrostatics does not provide the main contribution to the activation barrier. The dependence of the B-A transition center on the salt concentration is in the range expected from polyelectrolyte theory. An exchange of H_2_O against D_2_O did not induce changes of the equilibrium and kinetic parameters of the B-A transition within the limits of experimental accuracy. A comparison of the experimental results for the B-A transition with those for other stacking rearrangements indicates that the B-A activation barrier found at reduced water activity is on the expected order of magnitude, whereas the absence of a barrier indicated by MD simulations for the B-A transition in the absence of ethanol appears to be unusual from this point of view.
